# Growth, physiological, and temperature characteristics in chinese cabbage pakchoi as affected by Cd- stressed conditions and identifying its main controlling factors using PLS model

**DOI:** 10.1186/s12870-022-03966-2

**Published:** 2022-12-07

**Authors:** Lantao Li, Binglin Guo, Chenchen Feng, Haitao Liu, Di Lin

**Affiliations:** 1 College of Resources and Environment, Henan Agricultural University, 450002 Zhengzhou, China; 2 College of Forestry, Henan Agricultural University, No. 63 Nongye Road., Jinshui District, 450002 Zhengzhou, China

**Keywords:** Cadmium, Chinese cabbage pakchoi (*Brassica chinensis* L.), Physiological traits, Canopy temperature, PLS model

## Abstract

**Background:**

Although hormesis induced by heavy metals is a well-known phenomenon, the involved biological mechanisms are not fully understood. Cadmium (Cd) is a prevalent heavy metal in the environment. Exposure of Cd, via intake or consumption of Cd-contaminated air or food, poses a huge threat to human health. Chinese cabbage pakchoi (*Brassica chinensis* L.) is widely planted and consumed as a popular vegetable in China. Therefore, studying the response of Chinese cabbage pakchoi to Cd- stressed conditions is critical to assess whether cabbage can accumulate Cd and serve as an important Cd exposure pathway to human beings. In this study, we investigated the influence of Cd stress on growth, photosynthetic physiology, antioxidant enzyme activities, nutritional quality, anatomical structure, and canopy temperature in Chinese cabbage pakchoi. A partial least squares (PLS) model was used to quantify the relationship between physical and chemical indicators with Cd accumulation in cabbage, and identify the main controlling factors.

**Results:**

Results showed that Cd stress significantly inhibited cabbage’s growth and development. When Cd stress was increased, the phenotypic indicators were significantly reduced. Meanwhile, Cd stress significantly enhanced the oxidative stress response of cabbage, such as the activities of catalase (CAT), superoxide dismutase (SOD), peroxidase (POD), ascorbate peroxidase (APX), and the content of malondialdehyde (MDA) in leaves. Such a change tended to increase fenestrated tissues’ thickness but decrease the thickness of leaf and spongy tissues. Moreover, Cd stress significantly increased soluble sugar, protein, and vitamin C contents in leaves as well as the temperature in the plant canopy. The PLS model analysis showed that the studied phenotypic and physicochemical indicators had good relationships with Cd accumulation in roots, shoots, and the whole plant of cabbage, with high coefficient of determination (R^2^) values of 0.891, 0.811, and 0.845, and low relative percent deviation (RPD) values of 3.052, 2.317, and 2.557, respectively. Furthermore, through analyzing each parameter’s variable importance for projection (VIP) value, the SOD activity was identified as a key factor for indicating Cd accumulation in cabbage. Meanwhile, the effects of CAT on Cd accumulation in cabbage and the canopy mean temperature were also high.

**Conclusion:**

Cd stress has significant inhibitory effects and can cause damage cabbage’s growth and development, and the SOD activity may serve as a key factor to indicate Cd uptake and accumulation in cabbage.

**Supplementary Information:**

The online version contains supplementary material available at 10.1186/s12870-022-03966-2.

## Background

Soil pollution by heavy metals is a major global issue [1]. In recent decades, with the rapid urbanization and development of mining and metal smelting as well as the application of pesticides and fertilizers, heavy metals are continuously released into the environment, leading to their accumulation in the soil [2]. Among heavy metals, cadmium (Cd) is the most widely distributed pollutant in the environment, which threatens the health of plants and animals [3, 4]. Cd is a non-essential trace element and has several characteristics like strong toxicity, high mobility, great undegradability, concealment, etc. [5, 6] . Moreover, Cd can easily be absorbed and enriched by plants and further enter the food chain, threatening human health [7] . Previous studies have shown that Cd can induce a series of diseases when entering the human body, such as causing chronic toxicity and serious damage to kidneys and even neurological and immune systems [8]. Moreover, plants can also produce a series of stress responses to Cd contamination, such as oxidative stress response, imbalance of enzyme activities and plant signaling substances, which impair photosystems, alter enzyme activities, deteriorate quality, and damage cells, eventually affecting plant physiological and biochemical processes and inhibiting plant growth and development [9–11]. It is believed that a low concentration of Cd can cause acute damage to plant growth and development, like thin and dwarf plants, chlorotic leaves, and delayed fertility [12, 13]. This will ultimately lead to yield loss and quality deterioration, and further cause serious environmental and social problems. Therefore, Cd stress has become a major concern in the field of environmental pollution, and it is a burning issue to seek an effective strategy to mitigate Cd pollution in the environment.

Cadmium, with high biotoxicity, is an active heavy metal in natural ecosystems [14, 15]. When Cd enters the soil, it is mainly deposited in the topsoil and deteriorates soil quality, yet rarely migrates downward to the deep soil [16, 17]. In addition, Cd can easily become bioavailable and be absorbed by plant roots. Accumulation of Cd in plants significantly affects their normal growth and development [18]. To date, three main possible mechanisms were proposed to explain Cd toxicity in plants: (i) generation of reactive oxygen species; (ii) displacement of different proteins, including transcription factors and enzymatically active cofactors; and (iii) denaturation or inactivation of proteins by binding to free radicals [19, 20, 6]. When Cd accumulates in plant roots via cortical tissues to a certain amount, it will cause severe damage to root tip cells, inhibit their multiple enzyme activities, and reduce their nutrient uptake and transport capacity [21]. Meanwhile, when Cd is transported from roots to shoots, it will also generate seriously toxic effects (e.g., lipid peroxidation and enhanced malondialdehyde (MDA) generation) [22], which will also stimulate the plants’ antioxidant capacity in response to Cd stress and enhance their oxidative enzyme activities (e.g., superoxide dismutase (SOD), peroxidase (POD), and catalase (CAT) [23, 24]. Many previous studies revealed that Cd stress can directly or indirectly cause toxic effects on plants and disturb a series of physiological and biochemical processes in plants. Cd stress may also interfere with the uptake, utilization, and translocation of essential nutrients, inhibit photosynthetic pigment synthesis, and reduce the electron transfer in photosystem I [25–27]. Therefore, control of Cd contamination in the environment requires an in-depth understanding of how plants respond to Cd stress, including: (i) resolving the shift of plant growth, development, and physiological and biochemical characteristics under Cd stress; (ii) clarifying the plant’s response to Cd stress and the tolerance mechanism; and (iii) identifying the key influencing factor of Cd stress in plants.

At present, heavy metal contamination in vegetables has become a serious threat to food safety, and about 70% of Cd ingested by humans comes from edible vegetables [28, 29]. Among vegetables, leafy vegetables are widely grown and consumed in China. However, the Cd absorption and enrichment in leafy vegetables are extremely high, which are much higher than those of eggplant, beans, and root vegetables [30]. Thus, Cd contamination in leafy vegetables is a major concern. Chinese cabbage pakchoi (*Brassica chinensis* L.), a green leafy vegetable with good taste and low-calorie content, is widely grown in China and highly favored by consumers. Hence, to control the migration of Cd into the food chain and to ensure the quality and safety of agricultural products, it is of high importance to mitigate the Cd content in vegetables such as Chinese cabbage pakchoi. Unlike other plants [31–33], studies on Cd stress in Chinese cabbage pakchoi were rarely reported, and Chinese cabbage’s physiological and ecological responses to Cd stress are largely unknown. Moreover, the response of Chinese cabbage pakchoi to different Cd stress still needs to be further clarified in detail. Accordingly, this study was designed to systematically reveal the influence of Cd stress on Chinese cabbage pakchoi’s growth, photosynthetic physiology, antioxidant enzyme activities, nutritional quality, anatomical structure, and canopy temperature, through laboratory hydroponic experiments. A partial least squares (PLS) model was employed to quantify the relationship between physical and chemical indicators with Cd accumulation in Chinese cabbage pakchoi. Moreover, the main controlling factor for Cd toxicology and resistance in Chinese cabbage pakchoi was identified.

## Results

### The effects of cd stress on cabbage growth

**Fig. 1:**
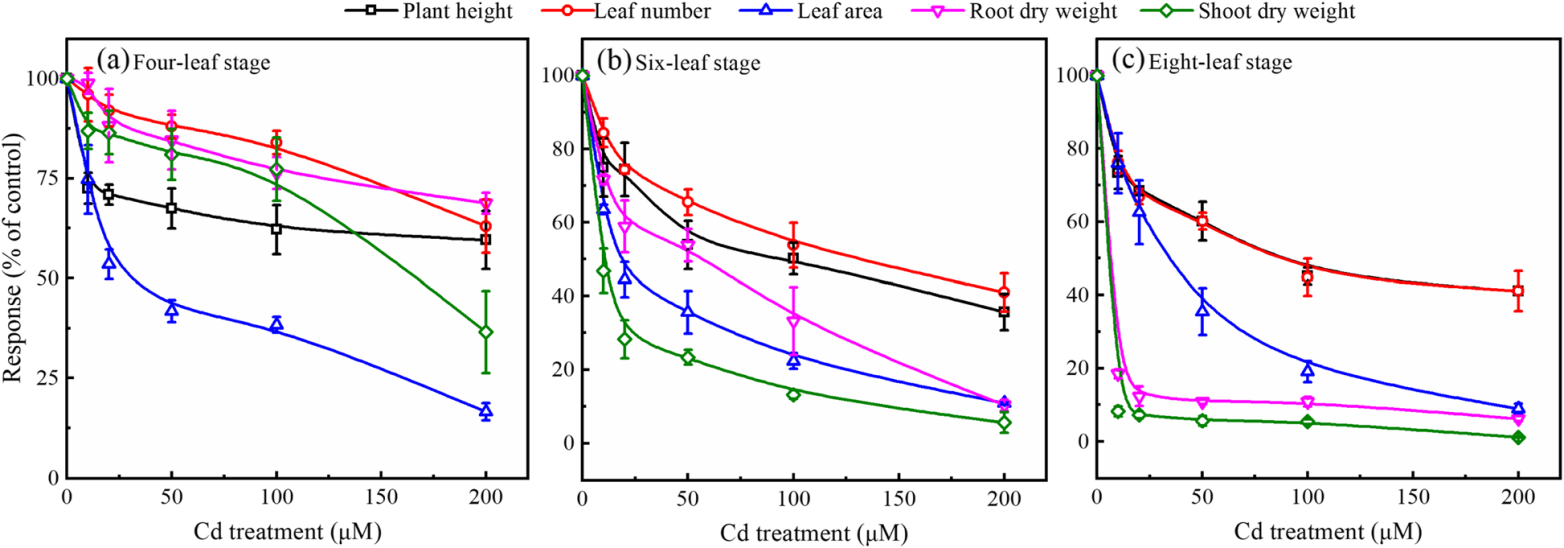
Effect of Cd stress on growth status in Chinese cabbage pakchoi treated with 0, 10, 20, 50, 100, and 200 μmol/L Cd at four-leaf, six-leaf and eight-leaf stage, respectively. Values are presented as mean ± standard deviation (*n* =5)

Cadmium stress significantly inhibited the growth and development of cabbage. The phenotypic indicators continuously decreased with increasing Cd concentrations in the hydroponic solution. Moreover, the inhibitory effect of Cd stress tended to increase with cabbage development (Fig. 1). Compared with the control (0 µmol/L Cd), plant height (-40.0%), leaf number (-31.3%), leaf area (-59.8%), root dry weight (-53.1%), and shoot dry weight (-66.3%) significantly decreased in the treatment groups.

### The effects of cd stress on cd accumulation in cabbage

**Fig. 2:**
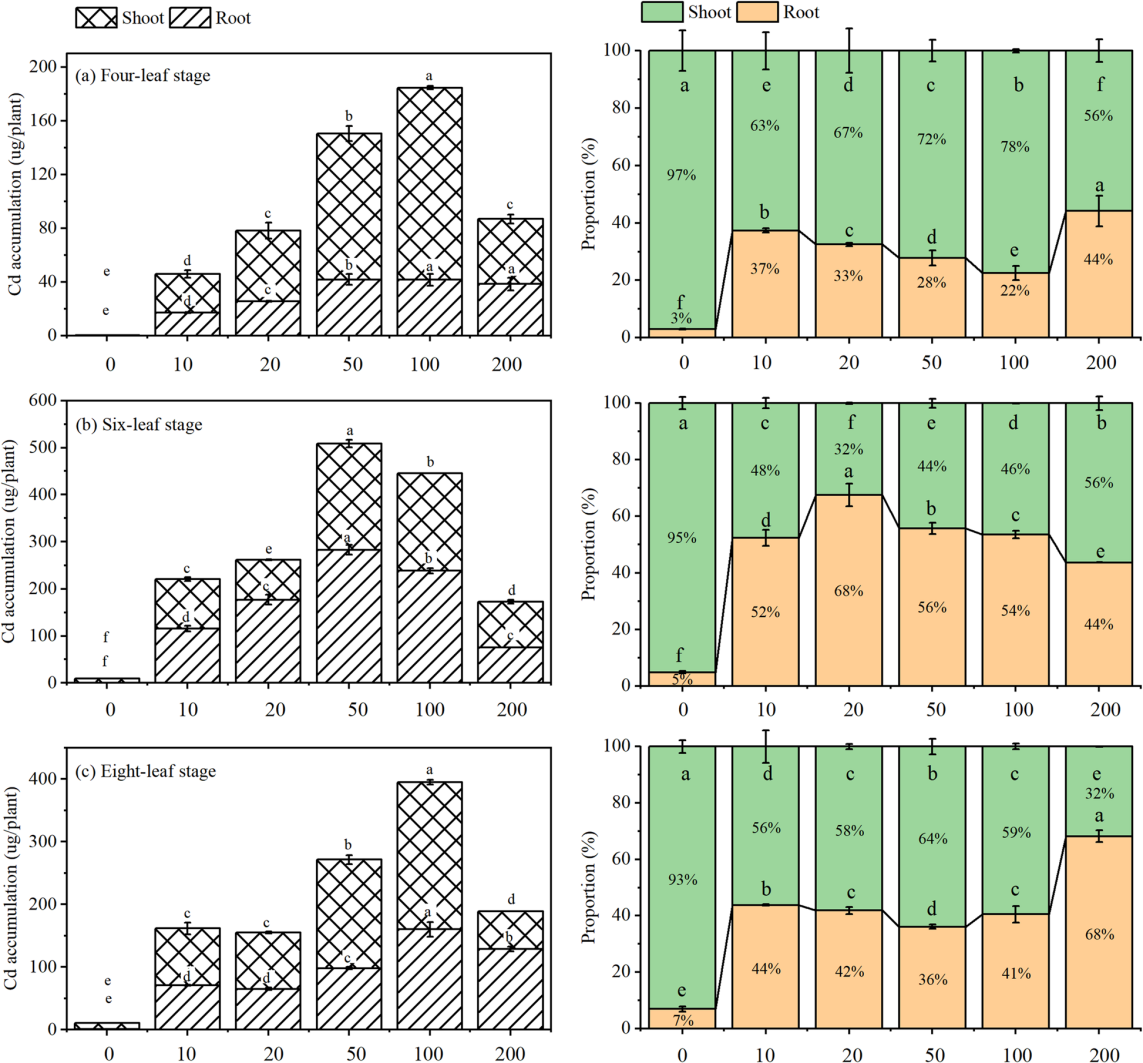
Effect of Cd stress on Cd accumulation (ug/plant) and proportion (%) of different parts (shoot and root) in Chinese cabbage pakchoi treated with 0, 10, 20, 50, 100, and 200 μmol/L Cd at four-leaf, six-leaf and eight-leaf stage, respectively. Values are presented as mean ± standard deviation (*n* =5). Different letters on the column represent significant differences in different treatments by LSD comparison at *P* <0.05

At different growth stages, Cd accumulation in cabbage roots and shoots showed a first increasing then decreasing trend with increasing Cd concentrations in the hydroponic solution, and a peak value of Cd accumulation (~ 100 µmol/plant) was observed under high Cd stress conditions. By contrast, Cd accumulation in shoots showed an obvious cumulative effect, with a value of 76.4, 144.0, and 129.9 µg/plant at the four-, six-, and eight-leaf stages, respectively. Similarly, the cumulative proportion of shoot Cd also showed a first increasing then decreasing trend when continuously lifting the Cd stress, and the highest value was observed under the Cd concentration of 100 µmol/plant (Fig. 2). This indicates that light to high Cd stress can enhance Cd accumulation in cabbage. However, when the Cd stress level exceeds the Cd tolerance range of cabbage, it can cause irreversible damage to the plant and lead to plant death.

### The effects of cd stress on photosynthetic parameters in cabbage

**Fig. 3:**
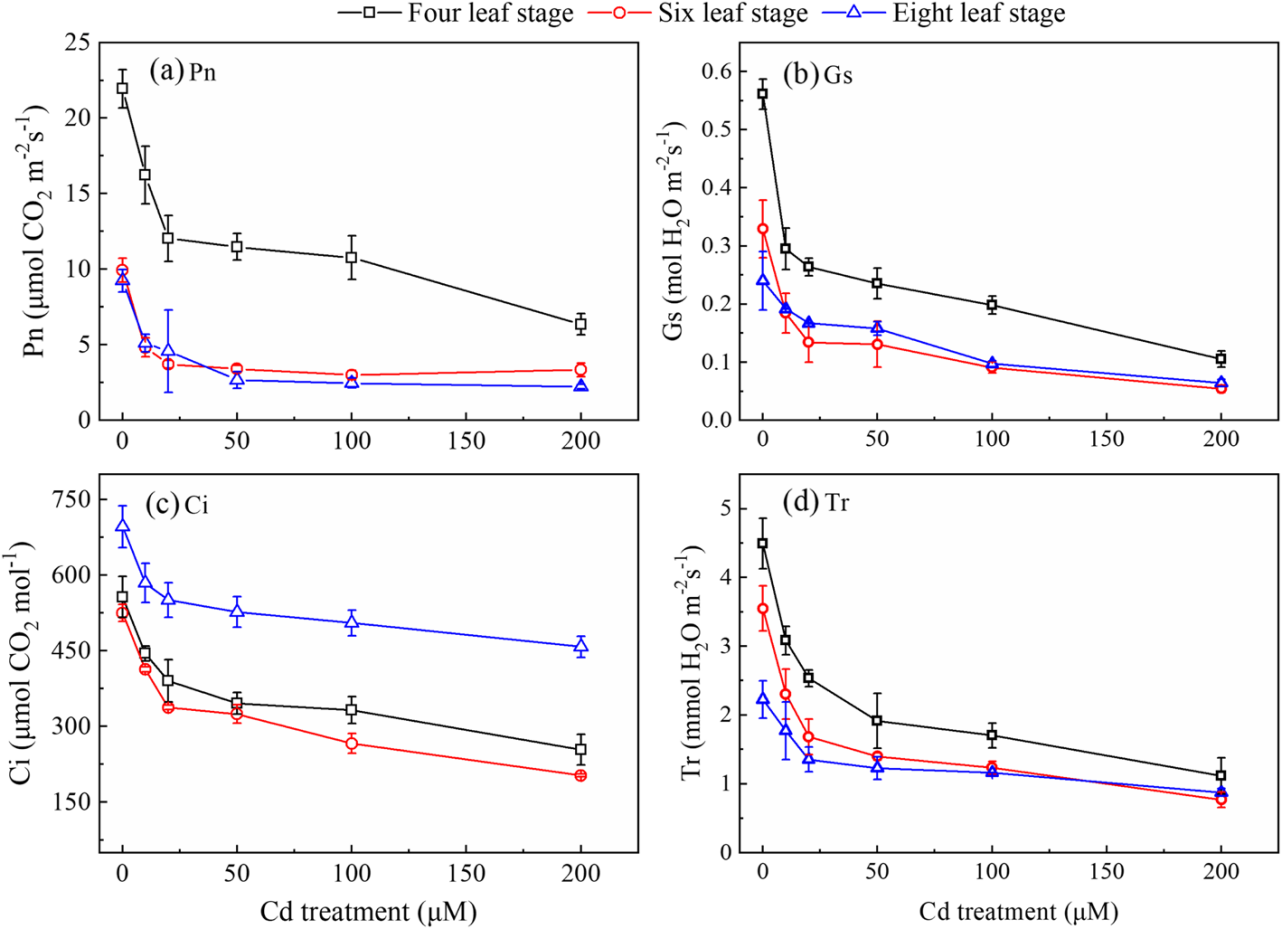
Effect of Cd stress on (a) photosynthetic rate (Pn), (b) stomatal conductance (Gs), (c) intercellular CO_2_concentration (Ci), (d) transpiration rate (Tr) in Chinese cabbage pakchoi treated with 0, 10, 20, 50, 100, and 200 μmol/L Cd at four-leaf, six-leaf and eight-leaf stage, respectively. Values are presented as mean ± standard deviation (*n* =5)

The photosynthetic parameters (e.g., Pn, Gs, Ci, and Tr) continuously decreased under low Cd stress; by contrast, the decrease of those parameters slowed down under high Cd stress, and a “plateau” state was finally obtained (Fig. 3). For Pn, its average value in the control (0 µmol/L) was 13.7 µmol·m^− 2^·s^− 1^, but only 3.1 µmol·m^− 2^·s^− 1^ in the treatment (200 µmol/L). Similarly, the average value of Gs in the control (0 µmol/L) and treatment (200 µmol/L) were 0.38 and 0.07 mol·m^− 2^·s^− 1^, respectively; and Ci and Tr in the control (0 µmol/L) and treatment (200 µmol/L) were 559.4 and 271.5 µmol/mol; and 3.42 and 0.92 mmol·m^− 2^·s^− 1^, respectively. The differences in the noted parameters between the control and treatment groups were significant.

### The effects of cd stress on antioxidant enzyme activities in cabbage

**Fig. 4:**
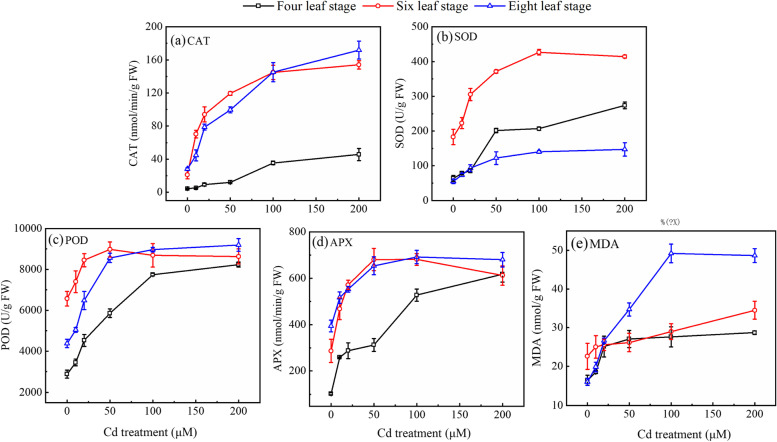
Effect of Cd stress on the activities of (a) CAT, (b) SOD, (c) POD, (d) APX and the content of (e) MDA in Chinese cabbage pakchoi treated with 0, 10, 20, 50, 100, and 200 μmol/L Cd at four-leaf, six-leaf and eight-leaf stage, respectively. Values are presented as mean ± standard deviation (*n* =5)

The antioxidant enzyme activity in plants is an important indicator to reflect the plant’s adaptation to exogenous stress. Compared with the control (0 µmol/L), the CAT, SOD, POD, APX activities and the MDA content in cabbage leaves significantly increased to a “plateau” state with the continuous increase of Cd concentrations, and the inflection point was 100 µmo/L Cd stress. The mean values of CAT were 17.9, 50.4, 77.0, 108.5, and 123.8 nmol/min/g for the control (0 µmol/L), low (10 and 20 µmol/L), medium (50 µmol/L), high (100 µmol/L), and severe (200 µmol/L) Cd stress, respectively. Moreover, the mean values of SOD were 100.3, 143.2, 231.6, 257.9, and 278.2 U/g in the noted groups, respectively; and the mean values of MDA were 18.3, 23.4, 29.3, 35.3, and 37.3 noml/g in the noted groups, respectively. In addition, other indicators such as POD and APX shared a similar trend (Fig. 4). These results indicate that exogenous Cd stress can effectively activate the antioxidant enzyme activities in cabbage plants and enhance their antioxidant response capacity.

### The effects of cd stress on quality indicators in cabbage

**Fig. 5:**
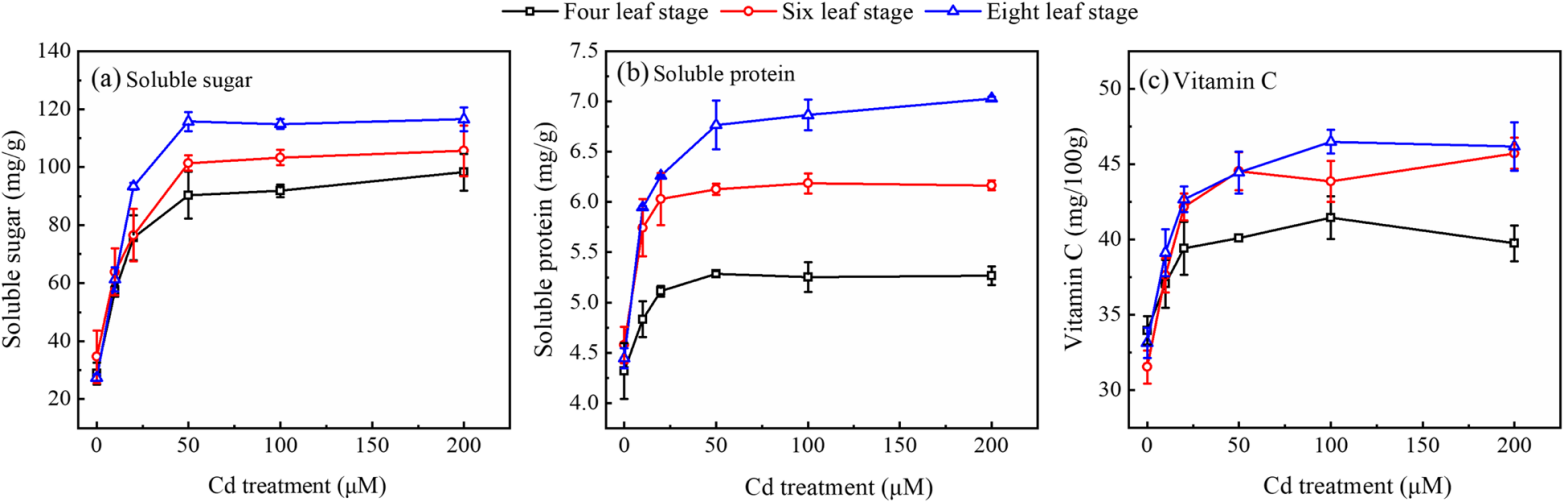
Effect of Cd stress on the content of (a) soluble sugar, (b) soluble protein and (c) vitamin C in Chinese cabbage pakchoi treated with 0, 10, 20, 50, 100, and 200 μmol/L Cd at four-leaf, six-leaf and eight-leaf stage, respectively. Values are presented as mean ± standard deviation (*n* =5)

Figure 5 clearly shows that exogenous Cd stress significantly increased the content of soluble sugar, protein, and vitamin C in cabbage. The average soluble sugar contents were 30.3, 60.8, 81.9, 102.5, 103.4, and 106.8 mg/g under 0, 10, 20, 50, 100, and 200 µmol/L Cd stress, respectively. The average vitamin C values under the noted 6 Cd stress were 32.9, 37.9, 41.4, 43.0, 43.9, and 43.9 mg/100 g, respectively.

### The effects of cd stress on anatomical characteristics in cabbage

Cd stress significantly affected the anatomical characteristics of cabbage leaves (Table 1). Under low Cd stress, the thickness of leaf and spongy tissues was higher, but the thickness of palisade tissues was lower, compared to the control. Under high Cd stress, the cell arrangement was not in order, resulting in a decreased cell gap. Meanwhile, the thickness of leaf and spongy tissues decreased, but the thickness of palisade tissues increased. These analytical results revealed that the average thickness of leaf, palisade, and spongy tissues were 293.8, 96.4, and 115.8 μm in the control (0 µmol/L), respectively; 271.9, 114.3, and 102.7 μm under low Cd stress (10 and 20 µmol/L), respectively; 254.8, 125.3, and 92.5 μm under moderate Cd stress (50 µmol/L), respectively; and 200.4, 140.4, and 70.6 μm under severe Cd stress (200 µmol/L), respectively, where the differences were further enhanced. In addition, the P/S (palisade vs.spongy tissues) ratio of cabbage also increased significantly from 0.85 to 0 µmol/L to 2.09 at 200 µmol/L (Table 1).

**Table 1:** Effect of Cd stress on the anatomical quantity characteristics in Chinese cabbage pakchoi treated with 0, 10, 20, 50, 100, and 200 µmol/L Cd at four-leaf, six-leaf and eight-leaf stage, respectively. Values are presented as mean ± standard deviation (*n* = 5). Different letters on the column represent significant differences in different treatments by LSD comparison at *P* < 0.05

Growth stage	Cd-treatment (µM)	Leaf thickness (µm)	Upper epidermal thickness (µm)	Lower epidermal thickness (µm)	Palisade mesophyll thickness (P) (µm)	Spongy mesophyll thickness (S) (µm)	P/S
Four- leaf stage	0	302.2a	18.4a	19.1a	87.0c	120.5a	0.73d
	10	260.3b	18.1a	17.6a	91.3c	95.4b	0.98 cd
	20	259.8b	17.7ab	15.1b	111.4b	93.8b	1.22c
	50	246.1b	15.5ab	14.6b	117.0b	93.4b	1.27c
	100	210.9c	15.0b	14.3b	122.0b	70.6c	1.74b
	200	169.4d	14.7b	11.4c	137.0a	56.4c	2.46a
Six- leaf stage	0	275.8a	26.6a	21.1a	100.3c	104.1a	0.99d
	10	274.1a	26.2a	20.6a	103.4c	102.1a	1.02 cd
	20	268.3a	24.5ab	19.3a	112.3bc	98.3ab	1.20bc
	50	253.9ab	24.0ab	16.6b	124.9ab	96.0ab	1.31b
	100	239.3bc	22.6ab	15.7b	127.9a	95.3ab	1.35ab
	200	228.1c	21.6b	12.1c	137.4a	90.4b	1.53a
Eight- leaf stage	0	303.3a	37.5a	29.2a	101.9c	122.7a	0.84e
	10	286.5b	31.5b	27.6a	128.2b	115.9a	1.12de
	20	282.3b	28.8b	19.2b	139.5ab	110.4a	1.27 cd
	50	264.3c	21.1c	17. 5bc	134.0ab	88.0b	1.53bc
	100	256.3c	19.9c	15.7c	143.5ab	77.4bc	1.92ab
	200	203.7d	16.2d	15.5c	146.7a	65.1c	2.29a

### The effects of cd stress on temperature characteristics in cabbage

**Fig. 6:**
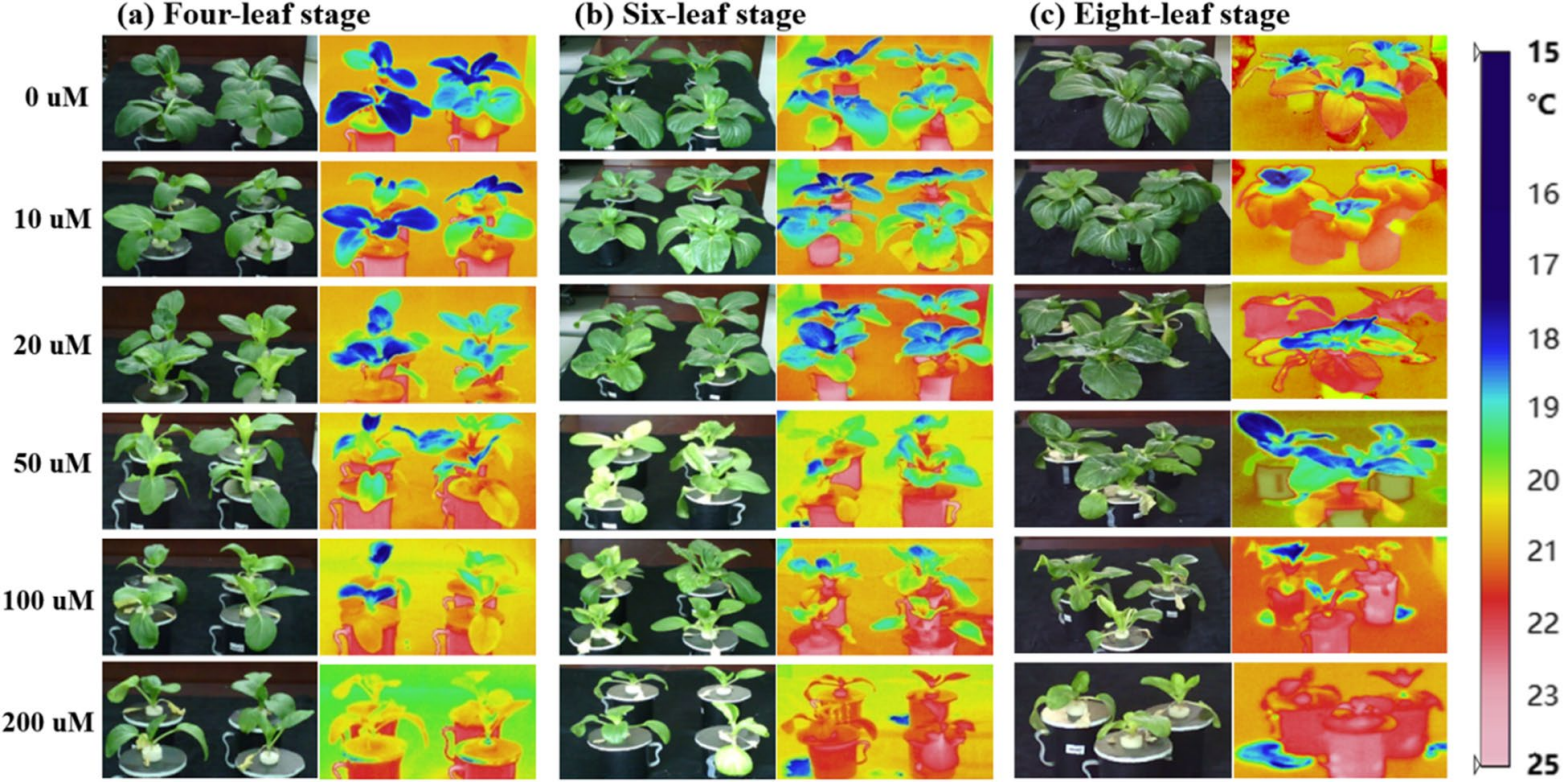
Effect of Cd stress on the infrared thermal image features in Chinese cabbage pakchoi treated with 0, 10, 20, 50, 100, and 200 μmol/L Cd at four-leaf, six-leaf and eight-leaf stage, respectively

When cabbage was subjected to exogenous Cd stress, it showed many changes: (i) plant was thin; (ii) leaves became wilted and yellowed; (iii) physiological characteristics were reduced; (iv) stomatal opening was days regulated; (v) transpiration was reduced. These changes could lead to an obvious increase in the temperature of the plant canopy (Fig. 6). shows that the mean canopy temperature of cabbage increased from 18.3, 20.5, and 18.7 °C in the control (0 µmol/L) to an average of 21.2, 23.3, and 20.3 ℃ in the treatment at the four-, six-, and eight-leaf stages, respectively. The differences were significant. Meanwhile, at different growth stages, changes in the maximum and minimum temperatures among different Cd treatments shared the same trend as the average temperature (Fig. 6).

**Table 2:** Effect of Cd stress on the plant temperature characteristics (maximum, minimum and average temperature) in Chinese cabbage pakchoi treated with 0, 10, 20, 50, 100, and 200 μmol/L Cd at four-leaf, six-leaf and eight-leaf stage, respectively. Values are presented as mean ± standard deviation (*n* =5). Different letters on the column represent significant differences in different treatments by LSD comparison at *P* < 0.05

Cd-treatment (µM)	Four-leaf stage			Six-leaf stage			Eight-leaf stge		
	Maximum	Minimum	Average	Maximum	Minimum	Average	Maximum	Minimum	Average
CK	20.0c	16.5c	18.3d	21.15d	19.8e	20.5a	16.9a	18.7bc	18.7bc
10 µM	20.6bc	17.2bc	18.9 cd	22.4c	18.8c	20.6d	21.0ab	16.2a	18.6bc
20 µM	20.6bc	18.0b	19.3bcd	22.1bc	19.0c	20.6d	20.7bc	17.3a	18.9b
50 µM	21.1ab	18.4b	19.8bc	22.7b	20.4b	21.5c	20.1bc	16.0a	18.1c
100µM	21.7a	18.4b	20.1b	23.9a	20.3b	22.1b	20.1bc	16.4a	18.3bc
200 M	22.0a	20.4a	21.2a	24.1a	22.5a	23.3a	21.0c	19.6b	20.3a

### Main controlling factors identified by the PLS model

#### Performance of the constructed PLS model

After clarifying the influence of exogenous Cd stression cabbage, a PLS model was used to analyze the relationship between the determined parameters with Cd accumulation in roots, shoots, and the whole plant at different growth stages (Table 2). The results illustrated that there was a good relationship between the physical and chemical parameters with Cd accumulation in different parts of cabbage during the reproductive growth period (R^2^> 0.80; RPD > 2.0). Hence, the constructed PLS model was very stable and accurate. In conclusion, the PLS-based quantitative analysis model constructed in this study is feasible to explain the regression relationship between different physicochemical parameters with Cd accumulation in cabbage, which is also helpful to reveal the key sensitive factor indicating the changes of Cd accumulation in cabbage.

**Table 3:** Accuracy evaluation of the PLS model describing the relationship between the above measured indicators and Cd accumulation in root, shoot and total in Chinese cabbage pakchoi

Model accuracy	Root Cd accumulation	Shoot Cd accumulation	Total Cd accumulation
R^2^	0.891	0.811	0.845
RMSE	26.483	30.922	56.438
RPD^a^	3.052	2.317	2.557

^a^RPD = the standard deviation /RMSE.

#### Main factors influencing Cd accumulation in cabbage

**Fig. 7:**
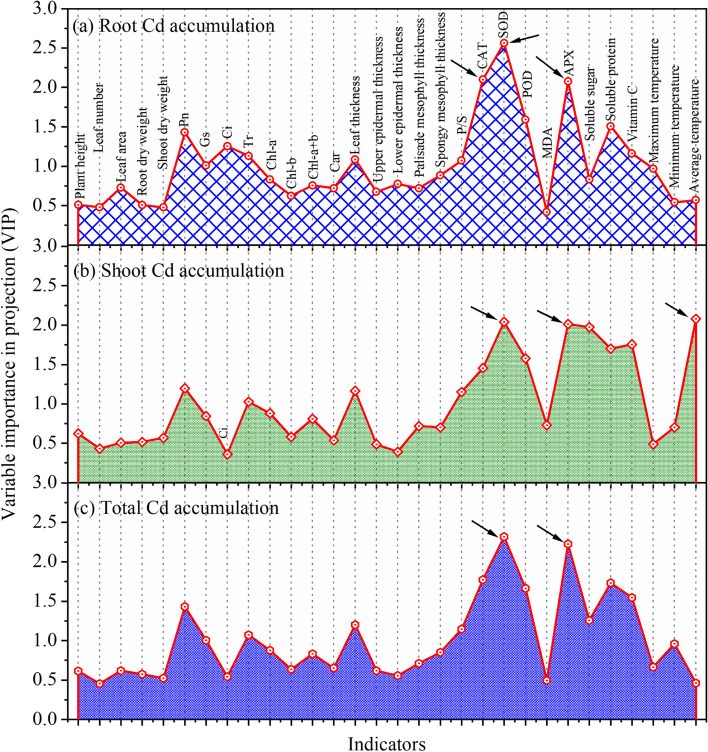
The VIP value for each indicator resulting from the PLS model and identifying the main controlling factors for (**a**) root Cd accumulation, (**b**) shoot Cd accumulation and (**c**) total Cd accumulation prediction in Chinese cabbage pakchoi

To further identify the main controlling factors influencing Cd accumulation in different tissues of cabbage, the VIP values of 30 physicochemical parameters vs. Cd accumulation in roots, shoots, and the whole plant were quantitatively calculated using the VIP analysis in the PLS model (Fig. 7). A high VIP value indicates that the tested parameter has a strong influence and high tightness. Based on these analyses, SOD was identified to be the most sensitive factor affecting the response of Cd accumulation in roots, shoots, and the whole plant, followed by APX. Meanwhile, CAT also had a strong influence on Cd accumulation in roots, and the average canopy temperature had a strong influence on Cd accumulation in shoots.

## Discussion

Cd is classified as a human carcinogen by the United Nations Environment Programme (UNEP) due to its high toxicity, widespread contamination, and high mobility in the natural environment. When excessive Cd is accumulated in plants, it will inhibit the root and plant growth [34]; disrupt cell membrane structure and function [35]; inhibit photosynthesis and respiration [36]; impair the synthesis of chlorophyll, soluble sugar, and protein [37]; reduce enzyme activities [38]; and induce oxidative stress in plants [39]. These negative influences ultimately lead to physiological/metabolic disorders and even the death of plants [40]. Many previous studies have shown that Cd stress can inhibit the growth of plant roots and stems, which can result in several outcomes: (i) dwarf plants; (ii) slow growth; (iii) leaf curling, deforming, and yellowing; and (iv) biomass drop [41]. Our results showed that Cd application reduced the growth of Chinese cabbage pakchoi, as evident from a significant decrease in plant phenotypic indicators (Fig. 1 and Fig. S2). The result in this study indicates that cabbage is very sensitive to Cd stress, and severe Cd stress can cause serious damage to the plant.

When roots absorb and accumulate a certain amount of Cd, it will not only significantly affect their metabolic activities but also inhibit the transport of nutrients from roots to shoots. Moreover, when Cd accumulates a certain amount in shoots, it will cause the destruction of chlorophyll systems, leading to a dramatic decrease in the photosynthetic rate and chlorophyll status [42, 4]. In addition, Cd stress was suggested to significantly influence the electron transfer in the mitochondrion and chloroplast, possibly via causing chloroplast swelling and deformation, disintegration of cystoid, increase of starch granules, and loose and disordered arrangement of mitochondrial basal lamellae in plants [11, 36]. It has also been pointed out that Cd stress can effectively inhibit photosynthetic processes such as light energy uptake, energy conversion, Rubisco’s enzyme carboxylation reaction, and CO_2_ diffusion in leaves (Sagardoy et al., 2010; Wi et al., 2020) [43, 44]. These may explain the rapid decrease in chlorophyll synthesis and the photosynthetic rate in plants under Cd stress. Here we found that photosynthetic indicators (i.e., Pn, Gs, Ci, and Tr) (Fig. 3) and chlorophyll contents (i.e., Chl-a, Chl-b, and Car) (Fig. S1) significantly decreased under Cd stress in cabbage, and the inhibitory effect tended to increase with increasing Cd concentrations. Our results are consistent with many previous findings in Indian mustard [45]  and wheat [46]. This is mainly due to the fact that Cd stress disrupts the balance between formation and removal of reactive oxygen species in photosystem I in chloroplasts, which results in a dramatic decrease in chlorophyll contents [47].

With regard to the antioxidant defense system in plants, abundant reactive oxygen radicals are generated when exposed to exogenous stress such as Cd. This will prompt the peroxidation of unsaturated fatty acids in the cell membrane, resulting in the formation of large amounts of MDA [48, 49]. Our results presented in this study (Fig. 4) are consistent with this theory. In addition, the protective enzyme system in plants also serves as an important defense mechanism for plants to cope with adverse stresses. The activated antioxidant systems (i.e., SOD, POD, CAT, and APX) are able to efficiently remove the free radicals produced by Cd stress in plants, preventing membrane lipid peroxidation. Enhancing antioxidant enzyme activity is an important strategy to alleviate stress in crops. The presence of exogenous Cd at a certain concentration in the growth medium may stimulate the activity of antioxidant enzymes to eliminate excessive ROS, such as H_2_O_2_, to improve plant growth [50] (Fig. 4 and Table S1). In peroxisomes purified from pea leaves, Cd produced an increase of the H_2_O_2_ content and imbalances in the activity of antioxidative enzymes [51]. Particularly, the formed superoxide radical (O2^•−^) is mainly scavenged by SOD, and H_2_O_2_ by POD and CAT. The changes in those enzymes’ activities can reflect the resistance of plants [52, 53] found that the elevated Cd stress enhanced SOD and POD activities in maize seedlings, but high concentrations of Cd inhibited APX activity. Similarly, we also found that SOD, POD, APX activities (Fig. 4) and H_2_O_2_ content (Table S1) in cabbage increased significantly with the increase in Cd concentrations, yet those indicators dropped to a plateau state under severe Cd stress (200 µmol/L). Our results are in line with the findings by Guo et al. (2019)[46] and Romero-Puertas et al. (2004) [54]  that Cd treatment effectively increased MDA and H_2_O_2_ and content as well as SOD, POD, and CAT activities in wheat leaves, favoring the detoxification of Cd contamination. Additionally, GSH and GSSG are important antioxidants which can maintain the intracellular redox balance and protect the plasma membrane from oxidation [55]. They are essential for the synthesis of phytochelatin which can form heavy metal- phytochelatin complexes and play an important role in the sequestration and segregation of heavy metal detoxification [56]. In our study, the GHS and GSSG content in Chinese cabbage pakchoi leaves were also increased significantly with the increase in Cd concentrations (Table S1). Furthermore, the increased content of GSH and GSSG content suggested that ROS is scavenged through the GSH cycle to protect plants from increasing concentration of Cd. Increased GSH and GSSG can also chelate Cd and be transported to vacuole to reduce Cd toxicity.

The anatomical structure of leaves shows the adaptation and evolution of the plant, reflecting the adaptation strategy to the environment [57]. Previous studies revealed that when plants suffer from exogenous stress such as pollution, drought [58], salt damage [59]  and other extreme weather conditions [60], their leaf anatomy shows significant stress responses, including increased palisade tissue thickness and reduced mesophyll thickness. In addition, plants can prompt their adaption to the external environmental changes via regulating their physiological and morphological structural features, such as mesophyll thickness and leaf epidermal tissues’ structure, reflecting the evolutionary adaptive responses to a specific habitat [61, 62]  reported that when plants were under the stress of exogenous pollutants, leaf cuticle and palisade tissues became thickened but spongy tissues became thinned. The increase of palisade tissue thickness can significantly improve the light energy utilization efficiency of the plant. This is mainly owing to the close relationship between crop leaf anatomy and their photosynthetic capacities [63]. In this study, we found that exogenous Cd stress significantly altered the leaf anatomical properties, i.e., the leaf thickness and spongy tissue thickness significantly decreased but the palisade tissue thickness significantly increased with increasing Cd concentrations (Table 1). This is in line with the previous findings [63]. In addition, we also identified that photosynthetic efficiency in cabbage leaves significantly decreased with increasing Cd concentrations. Changes in photosynthetic efficiency in the crop are the most critical factor affecting crop biosynthesis and development, which are closely related to crop canopy temperature [64]. Higher temperatures may enhance crop growth, mainly through extending the growing season. However, extreme high temperature can cause cell damage and death and decreased growth as well as affecting quality, production, and photosynthetic efficiency [65, 60] . Therefore, the appropriate temperature is the key to maintain the normal metabolic development and photosynthesis of the crop [66]. In contrast, either too high or too low temperature is detrimental to the biosynthesis and development of the crop [67]. Our results showed that with increasing Cd concentrations, the physicochemical indicators of Chinese cabbage pakchoi were significantly inhibited. Then respiration and transpiration were dramatically influenced. As a result, the mean canopy temperature of cabbage significantly increased with increasing Cd stress (Fig. 6 and Table 2 ). Previous studies reported that Gs allows the exchange of CO_2_ and H_2_O, accompanied by photosynthesis and transpiration. Cd can regulate Gs to increase transpirational water loss from leaves under Cd stress conditions. The flow of water by transpiration drives the transport rates of nutrients, while the evaporative water loss from the plant surface removes the heat energy dissipated from absorbed but unconverted light during photosynthesis. The removal of heat energy increases with the transpiration rate (Tr), which reduces the temperature of the photosynthetic organ. This is the underlying mechanism of the decrease in canopy temperature in cabbage under Cd stress.

The PLS modeling results showed that the physical and chemical indicators of cabbage had good quantitative regression relationships with Cd accumulation in the root, shoot, and whole plant (Table 3). This indicates that all the variables have a similar response to Cd stress. In addition, the quantitative analysis of VIP-PLS showed that SOD is the most critical factor affecting Cd accumulation in all parts of cabbage (Fig. 7). SOD is believed to be the main enzymatic antioxidant defense system of plants. It usually serves as the first defense line to remove the abundant reactive oxygen species produced by plants under Cd stress. SOD is known to catalyze the dismutation of superoxide, which can effectively protect plant cells and organs by enhancing the tolerance of plants to Cd stress [68, 69]. Previous reports illustrated that when plants are under heavy metal stress, they will produce oxidative stress response, resulting in the accumulation of considerable reactive oxygen species in plant cells. On the one hand, oxidative stress can cause the peroxidation of membrane lipids. This will directly affect the integrity of the membrane, induce damages in DNA, proteins, lipids, and other functional macromolecules, and may even lead to apoptosis. On the other hand, oxidative stress can inhibit photosynthesis. Heavy metal stress tends to induce reduced photosynthesis and even wilting or death in plants. SOD plays a critical role in scavenging reactive oxygen species in plants, and oxidative stress of plants can be further alleviated by the involvement of other antioxidant enzymes. When exposed to exogenous stress, the antioxidant enzyme activity can be regulated to some extent by applying certain exogenous substances, which is quite helpful to enhance plant resistance [70–72].Therefore, the role of SOD in the defense response of plants to Cd is crucial. Enhancement of SOD activity in cabbage through certain measures is promising to improve plant tolerance to Cd. The relative research will provide novel insights for bioremediating Cd-contaminated soil.

## Conclusion

In this study, we systematically investigated the influence of Cd stress on cabbage growth, photosynthetic physiology, antioxidant enzyme activities, nutritional quality, anatomical structure, and canopy temperature. The results showed that Cd stress could significantly affect the growth and development of cabbage. With increasing Cd concentrations, the phenotypic indicators were significantly reduced. Cd stress significantly inhibited photosynthetic/ physiological processes in cabbage and simultaneously reduced chlorophyll contents and photosynthetic rates in leaves. In addition, with the increase of Cd stress, the oxidative stress response in cabbage was stimulated. Leaf CAT, SOD, POD, APX activities and MDA, H_2_O_2_, GSH and GSSG content were significantly increased. In terms of leaf anatomy, Cd stress significantly reduced the thickness of leaves and spongy tissues but increased the thickness of palisade tissues. The PLS modeling illustrated that SOD was the key controlling factor for the change of Cd accumulation in roots, shoots, and the whole plant. Meanwhile, the effect of CAT on Cd accumulation in roots was quite high, which was also true for the influence of mean canopy temperature on Cd accumulation in shoots. Our findings in this study provide important theoretical support and references for the mechanical elucidation of Cd stress’ influence on cabbage physiology, and point out the direction for developing an efficient and eco-friendly method to remediate soil Cd pollution.

## Materials and methods

### Experimental materials and design

Chinese cabbage pakchoi (*Brassica chinensis* L.) seeds were purchased from Shanghai ShenGeng agricultural development Co., Ltd. The purchased cabbage has the following characteristics: total growth period of about 150 days; highly adaptable across China; good quality; 30 cm tall; 30 cm spreading; about 500 g per plant; high yield (about 4000 kg per 666.7 m^2^).

The experimental research and field studies on plants (either cultivated or wild), including the collection of plant material, are comply with relevant institutional, national, and international guidelines and legislation. Hydroponic experiments were carried out in the hydroponic laboratory of Henan Agricultural University. Before the experiment, cabbage seeds were soaked in 15% H_2_O_2_ for 15 min, washed with distilled water, soaked in warm water at 30 ℃ for 20 min, and then evenly placed in a special seedling tray with a small amount of water. The seeds were firstly cultured in an incubator under constant temperature (i.e., 25 ℃) for 120 h, and then transferred to an artificial climate chamber. When cabbage seeds grew to the two-leaf-and-a-bud stage, identical seedlings were selected and transplanted into 1/8 Hoagland nutrient solution in black plastic boxes for the following cultivation. The plastic boxes were covered with black foam plates with small uniform holes, which are able to hold the planted seedling well. During the cultivation, the nutrient solution was gradually adjusted to 1/4-, 1/2-, and full-strength Hoagland solution at an interval of 3 days. The hydroponic experiments were performed in an artificial climate chamber with 16 h/ 8 h light/dark conditions. The temperature and relative humidity were maintained at 20 ℃ and 55-65%, respectively.

When the seedling grew to the three-leaf stage, full-strength Hoagland solution with 6 Cd concentrations (0, 10, 20, 50, 100, and 200 µmol/L, Cd in the form of CdCl_2_·2.5 H_2_O) was utilized. Low (10 and 20 µmol/L), medium (50 µmol/L), high (100 µmol /L), and severe (200 µmol/L) Cd stress were systematically considered in this study. Hoagland solution was replaced every 3 days, while the solution pH was controlled around 6.0 during the cultivation (Fig. S2). Each treatment had five replicates.

### Data acquisition

#### Growth indicators

The plant height of cabbage was measured at four-, six-, and eight-leaf stages using a ruler to an accuracy of 0.1 cm. Leaf area was measured non-destructively using a digital camera [73]. The above- and below-ground fresh biomass were weighed firstly and then dried in an oven at 105 °C for 30 min to stop the cell metabolism. The plant tissues were then dried at 70 °C until a constant weight, and weighed.

#### Determination of photosynthetic parameters

Photosynthetic activities were measured at four-, six-, and eight-leaf stages, using a LI-6800 photosynthesis measurement system. The measurement was performed on the first fully expanded leaf. The parameters included photosynthetic rate (Pn), stomatal conductance (Gs), intercellular CO_2_ concentration (Ci), and transpiration rate (Tr). The light intensity was set at 1200 µmol·m^− 2^·S^− 1^, and a CO_2_ buffer cylinder was adopted to ensure a stable gas flow.

#### Thermal imaging assessment of the plant

To investigate the potential influencing mechanism of Cd on cabbage growth and development, a Fluke TiX650 infrared thermal camera was further used to test the canopy temperature under different Cd stress. The camera has a spatial resolution of 0.87 mRad (i.e., 640 × 480 pixel focal plane array and 32 × 24 field of view lens). The thermal resolution of the camera reaches 0.025 ℃ at an ambient temperature of 30 ℃. During measurement, the camera was fixed at a height of 1.0 m above the canopy, and a photographed observation angle of 45° was used. Four photographs were taken for each replicate, and the canopy temperature was calculated and analyzed using SmartView software.

#### Cd concentration and accumulation in cabbage

Approximately 10.0 mg of dried and ground plant samples were weighed and placed into a digestion tube. The sample was digested with a mixture of concentrated HNO_3_ and HClO_4_ (3:1, v/v). The Cd concentration in the digestion solution (µg/L) was analyzed by an atomic absorption spectrophotometer (AAS, ZEEnit70d0, Analytikjena, Germany). And then, Cd concentration (µg/kg) and accumulation (µg/plant) in different tissues of cabbage (i.e., roots and shoots) were calculated.

#### Determination of chlorophyll contents and antioxidant enzyme activities

The fully expanded leaves of cabbage were collected in each sampling. The chlorophylls were extracted with acetone. Chlorophyll contents (i.e., Chl-a, Chl-b, and Car) in the extract were measured by the spectrophotometric method; CAT activity by the iodometric method; SOD activity by the Azo blue tetrazole method; POD activity by the guaiacol method; and MDA content by the thiobarbituric acid method. The H_2_O_2_ content was measured with the method of Sergiev et al. [74], while glutathione (GSH) and oxidized glutathione (GSSG) content was determined following the method of Murshed et al. [75]. In addition, the determination of ascorbate peroxidase (APX) activity followed the method proposed by Nakano and Asada [76].

#### Determination of quality indicators

After the measurement of physiological and biochemical parameters in cabbage, fresh leaves were collected for quality indicator determination. The soluble sugar, protein, and vitamin C contents were analyzed by the anthrone colorimetric method, the Coomassie blue method, and titration with 2-6-dichlorophenol-indophenol, respectively.

### Anatomical analysis

During each sampling, the first fully expanded leaf of cabbage was selected. A small piece of leaf with a size of 1 cm×3 cm was cut at about 0.5 cm apart from the main vein and immediately fixed and embedded to make transverse leaf sections, which were dyed with toluidine blue. The slices were photographed by an OLYMPUS BH2 microscope with a 200x lens, and five views were selected for each sample. A series of parameters (including leaf thickness, upper epidermal thickness, lower epidermal thickness, palisade mesophyll thickness, and spongy mesophyll thickness) was measured using Image-Pro Plus 6.0 software. The effects of Cd stress on the anatomical structure of cabbage leaves were analyzed.

### Statistical analyses

After clarifying the characteristics of Cd accumulation (a dependent variable) and cabbage’s physicochemical indicators (independent variables, 30 variables in total) under different Cd stress, a PLS model was employed to quantify the relationship between the two kinds of variables, and the key factor affecting the change of Cd accumulation was identified. By combining the PLS model with classical correlation analysis, multiple linear regression analysis, and principal component analysis, the dimensionality of factor analysis will be greatly reduced, which will also be helpful to reveal the key controlling factor from multiple variables. We then constructed a highly robust analysis model, following a previous report [77]. The coefficient of determination (R^2^), root mean square error (RMSE), and relative percent deviation (RPD) between the observed and predicted values were used to assess the performance of the PLS model. Higher R^2^ and RPD and lower RMSE values indicate a more stable and accurate model. The variable importance for projection (VIP) in the PLS model was adopted to identify the key controlling factor. This is because VIP can quickly, intuitively, and quantitatively reflect the importance of each variable in its prediction of the dependent variable. When VIP approaches 1.0, the analysis indicates that the variable is more sensitive and may pose a big influence [78].

The raw data input and analysis were performed in Excel 2007 software. Analysis of variance (ANOVA) was performed by SPSS 20.0 software. Quantitative assessment of the model accuracy and selection of key factors were achieved using the PLS plug-in unit in Matlab R2012a. All figures were plotted by Origin 2019 software.

## Supplementary Information

**Additional file 1:** **Fig S1. **Effect of Cd stress on (a) Chl-a, (b) Chl-b, (c) Chl-a+b and (d) Car content in Chinese cabbage pakchoi leaves treated with 0, 10, 20, 50, 100, and 200 μmol/L Cd at four-leaf, six-leaf and eight-leaf stage, respectively. Values are presented as mean ± standard deviation (*n* =5). **Fig S2.** Photographs of Chinese cabbage pakchoi under different Cd stress conditions. **Table S1.** Effect of Cd stress on H_2_O_2_, GSH and GSSG content in Chinese cabbage pakchoi leaves treated with 0, 10, 20, 50, 100, and 200 μmol/L Cd at four-leaf, six-leaf and eight-leaf stage, respectively. Values are presented as mean ± standard deviation (*n* =5). Different letters on the column represent significant differences in different treatments by LSD comparison at *P* <0.05.

## Data Availability

All data generated or analyzed during this study are included in this article and available from the corresponding author on reasonable request, and all data during this study are included in this published article and its supplementary information files.
