# Supplementary material for: Growth, physiological, and temperature characteristics in chinese cabbage pakchoi as affected by Cd- stressed conditions and identifying its main controlling factors using PLS model
Source: BMC Plant Biol. 2022 Dec 7;22:571. doi: 10.1186/s12870-022-03966-2 (PMC9727860; doi:10.1186/s12870-022-03966-2)
Supplement: Supplementary file 1 — Additional file 1: Fig S1. Effect of Cd stress on (a) Chl-a, (b) Chl-b, (c) Chl-a+b and (d) Car content in Chinese cabbage pakchoi leaves treated with 0, 10, 20, 50, 100, and 200 μmol/L Cd at four-leaf, six-leaf and eight-leaf stage, respectively. Values are presented as mean ± standard deviation (n =5). Fig S2. Photographs of Chinese cabbage pakchoi under different Cd stress conditions. Table S1. Effect of Cd stress on H2O2, GSH and GSSG content in Chinese cabbage pakchoi leaves treated with 0, 10, 20, 50, 100, and 200 μmol/L Cd at four-leaf, six-leaf and eight-leaf stage, respectively. Values are presented as mean ± standard deviation (n =5). Different letters on the column represent significant differences in different treatments by LSD comparison at P <0.05. [file 12870_2022_3966_MOESM1_ESM.docx]

**Supplementary material for**

**Growth, physiological, and temperature characteristics in Chinese cabbage pakchoi as affected by Cd- stressed conditions and identifying its main controlling factors using PLS model**

|  |
| --- |
| **Fig. S1.** Effect of Cd stress on (a) Chl-a, (b) Chl-b, (c) Chl-a+b and (d) Car content in Chinese cabbage pakchoi leaves treated with 0, 10, 20, 50, 100, and 200 μmol/L Cd at four-leaf, six-leaf and eight-leaf stage, respectively. Values are presented as mean ± standard deviation (n =5). |

| **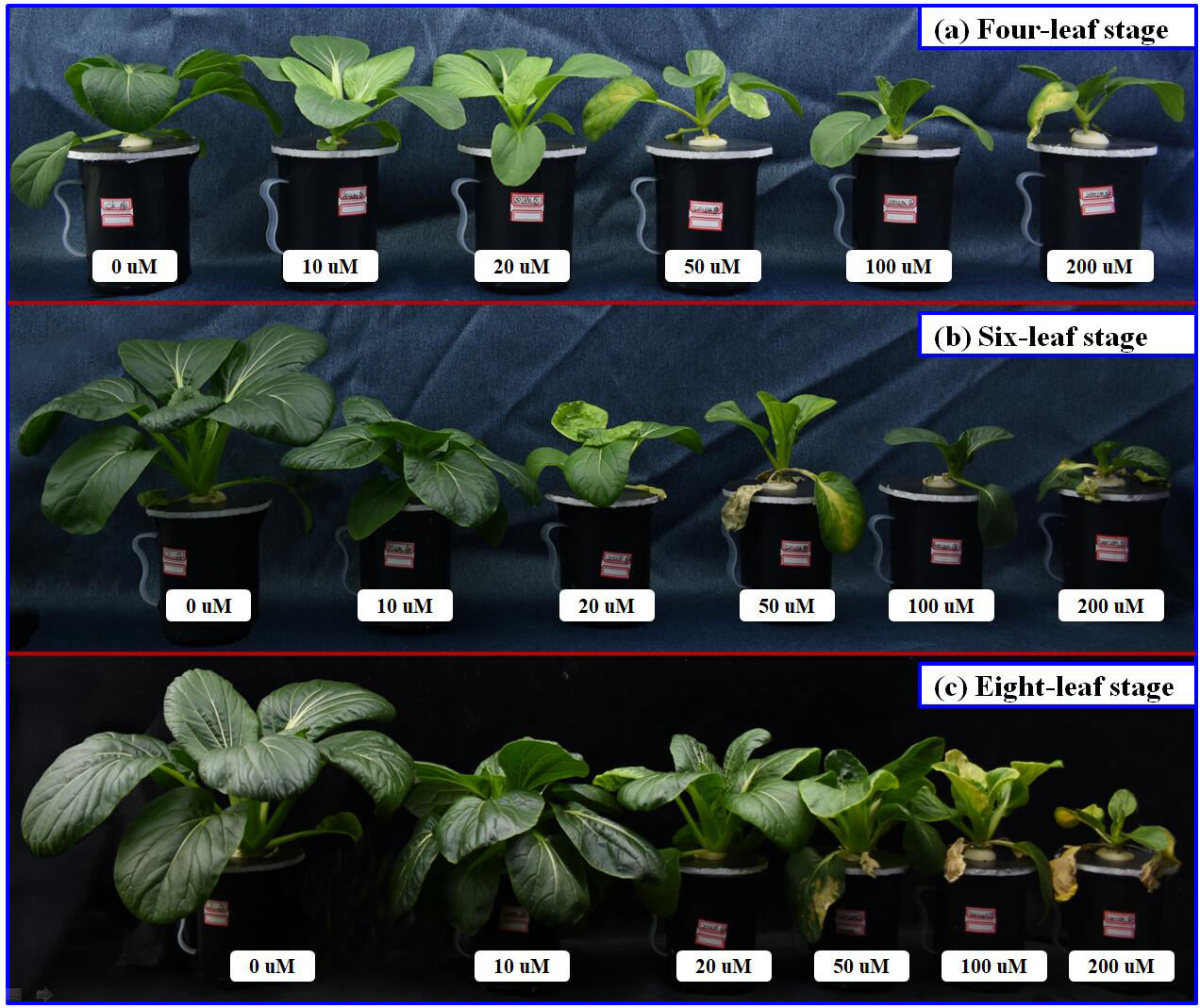** |
| --- |
| **Fig. S2.** Photographs of Chinese cabbage pakchoi under different Cd stress conditions. |

**Table S1**

Effect of Cd stress on H_2_O_2_, GSH and GSSG content in Chinese cabbage pakchoi leaves treated with 0, 10, 20, 50, 100, and 200 μmol/L Cd at four-leaf, six-leaf and eight-leaf stage, respectively. Values are presented as mean ± standard deviation (n =5). Different letters on the column represent significant differences in different treatments by LSD comparison at *P* <0.05.

| Cd-treatment (μM) | Four-leaf stage | | | Six-leaf stage | | | Eight-leaf stge | | |
| --- | --- | --- | --- | --- | --- | --- | --- | --- | --- |
|  | H_2_O_2_ (umol/g) | GSH (ug/g FW) | GSSG (ug/g FW) | H_2_O_2_ (umol/g) | GSH (ug/g FW) | GSSG (ug/g FW) | H_2_O_2_ (umol/g) | GSH (ug/g FW) | GSSG (ug/g FW) |
| CK | 19.34e | 24.33e | 31.73e | 11.45e | 30.06e | 37.34e | 13.75e | 26.78e | 21.71f |
| 10 μM | 25.61de | 29.09e | 38.36de | 16.53de | 34.76e | 43.57de | 18.25d | 30.46de | 29.69e |
| 20 μM | 29.47cd | 36.25d | 45.48cd | 20.31cd | 41.68d | 50.18cd | 22.17c | 34.30cd | 37.22d |
| 50 μM | 34.39bc | 43.85c | 51.39bc | 25.78bc | 49.35c | 56.63bc | 26.15b | 39.08c | 44.27c |
| 100μM | 38.90ab | 51.61b | 56.34ab | 30.31ab | 58.40b | 63.14b | 29.24b | 46.87b | 51.82b |
| 200 M | 43.74a | 62.14a | 64.48a | 33.54a | 66.42a | 71.88a | 34.41a | 54.51a | 58.60a |
